# Effectiveness of Persian Golnar on Excessive Menstrual Bleeding in Women with Abnormal Uterine Bleeding, Compared to Tranexamic Acid: A Triple-Blind, Randomized Equivalence Trial

**DOI:** 10.1155/2023/5355993

**Published:** 2023-07-20

**Authors:** Somayeh Es-haghee Ashteany, Marzieh Vahid Dastjerdi, Malihe Tabarrai, Fatemeh Nejatbakhsh, Seyede Nargess Sadati Lamardi, Azam Rahmani, Mohammad Azizkhani, Zahra Tavoli

**Affiliations:** ^1^Department of Traditional Medicine, School of Persian Medicine, Tehran University of Medical Sciences (TUMS), Tehran, Iran; ^2^Department of Obstetrics and Gynecology, Tehran University of Medical Science, Tehran, Iran; ^3^Food Microbiology Research Center, Tehran University of Medical Sciences, Tehran, Iran; ^4^Department of Traditional Pharmacy, School of Persian Medicine, Tehran University of Medical Sciences, Tehran, Iran; ^5^Nursing and Midwifery Care Research Center, School of Nursing and Midwifery, Tehran University of Medical Sciences, Tehran, Iran; ^6^Department of Obstetrics and Gynecology, Ziaeian Hospital, Tehran University of Medical Science, Tehran, Iran

## Abstract

**Introduction:**

Abnormal uterine bleeding (AUB) is a major healthcare problem in females of reproductive age and impacts women's health and quality of life (QoL). This study aimed to test the equivalence of Persian Golnar (PG) and tranexamic acid (TA) for the treatment of excessive menstrual bleeding.

**Method:**

A triple-blind randomized equivalence trial with parallel design and block randomization technique was performed. A total of 80 patients with AUB were randomly allocated to receive either PG or TA for three consecutive menstrual cycles. Blood loss was measured by the Pictorial Blood Loss Assessment Chart (PBAC). Hematological evaluations were done before the intervention and after treatment. QoL and premenstrual dysphoric disorder (PMDD) as secondary outcomes were assessed using the menorrhagia questionnaire (MQ) and Premenstrual Symptoms Screening Tool (PSST). Statistical analysis was performed using an independent *t*-test, paired *t*-test, *χ*2 test, Mann–Whitney test, and Wilcoxon signed-rank test.

**Results:**

Seventy-six women completed the 12-week follow-up. Both PG and TA groups experienced a significant reduction in blood loss. Furthermore, the serum level of hemoglobin in the PG group enhanced significantly (*P* < 0.001). QoL and PMDD scores were significantly improved in both groups (*P*< 0.001).

**Conclusion:**

The findings of the current trial supposed that the Golnar product is as effective as tranexamic acid in controlling bleeding and enhancing the quality of life and premenstrual symptoms.

## 1. Introduction

Abnormal uterine bleeding (AUB) is a wide term that incorporates any disorder in the cycle of menstruation, like volume, frequency, irregularity, and duration [1]. It is one of the most common gynecological issues, so at least about 30% of women of childbearing age deal with this problem [2]. It not only lowers patients' quality of life but also leads to a heavy financial burden, reduction of productivity rate, etc. [3, 4]. The causes of AUB are classified into two main categories of structural, anatomic uterine defects like polyp, adenomyosis, leiomyoma, malignancy, and hyperplasia, and non-structural, such as coagulopathy, ovulatory dysfunction, endometrial disorders, and iatrogenic [5].

Several conventional treatments are available for controlling bleeding, such as combined oral contraceptives, progestogens, non-steroidal anti-inflammatory drugs (NSAIDs), tranexamic acid, and surgery [6]. Nevertheless, a number of factors, like etiology, bleeding acuity, underlying comorbidities, fertility desire, expenses, potential side effects, and efficacy, should be taken into account over treatment [7]. Hormonal treatments, injection or oral, are considered the first line in AUB management, particularly in acute type [8], although they could not be used in the long term due to side effects [9]. Non-hormonal medications like tranexamic acid, as an antifibrinolytic agent, can decrease blood loss by half in menstruation and even improve quality of life; however, its efficacy is less in some cases, like uterine fibroids, and may cause some side effects like gastrointestinal disturbances, headache, anemia, fatigue, and thrombosis [8, 10].

Nowadays, many people and even physicians have a tendency toward complementary and alternative medicine (CAM), due to concerns about potential side effects and inadequate efficacy of conventional medicines [11]. There is a wide variety of CAM modalities in different cultures, but herbal medicine is the most popular one [11]. Many medicinal plants have been presented for the treatment of gynecological conditions, such as irregularity in menstruation, dysfunctional uterine bleeding, oligomenorrhea, and amenorrhea [12, 13]. For example, the results of a meta-analysis demonstrated that Chinese herbal medicines could have more benefits in the normalization of menstruation, menstrual symptoms, and hemoglobin count, with lower complications compared to Western medicine [14]. Moreover, Traditional Persian Medicine (TPM), as a modality of CAM, has been used for thousands of years and introduces several herbs like myrtle, ginger, and pomegranate flower to control excessive menstrual bleeding [15–17].

Based on TPM texts, a polyherbal composition, including pomegranate flower, myrtle, and gum Arabic, could be effective in the treatment of heavy bleeding. Pomegranate flower (*Punica granatum* Linn.), also known as Persian Golnar (PG), is a herbal medicine claimed to have antihemorrhagic effects and is used in conditions like abnormal uterine bleeding in TPM [18]. Antibleeding effects of pomegranate products have been shown in different disorders including epistaxis, gastrointestinal bleeding, hematemesis, and hemorrhoids [19]. Moreover, some clinical trials reported that pomegranate flower can also be effective in the reduction of menorrhagia [18, 20, 21]. These effects may be due to the presence of astringent agents such as tannins and triterpenoids and antioxidant and anti-inflammatory compounds that suppress the synthesis of prostaglandins [22]. In addition, it has been shown that myrtle (*Myrtus communis* L.), which is an evergreen shrub that grows in Iran, has antiseptic, antioxidant, anti-inflammatory, ulcer protective, and astringent properties [23].

Regarding previous findings, this study was designed to investigate whether the Golnar product is equivalent to tranexamic acid (TA) in reproductive women with heavy uterine bleeding in a 3-month duration of treatment.

## 2. Methods

### 2.1. Trial Design

A 12-week, single-center, randomized, triple-blind, equivalence trial was carried out in the Arash Hospital affiliated with the Tehran University of Medical Sciences between September 2018 and March 2020.

The protocol of the trial was approved by the Review Board and the Ethical Committee of Tehran University of Medical Sciences (number: IR.TUMS.VCR.REC.1397.144) and followed the Declaration of Helsinki and its subsequent revisions. The trial was registered at the Iranian registry of clinical trials with the code of IRCT20180622040186N1 on June 26, 2018 (https://en.irct.ir/trial/32084). A written consent form was taken from all eligible participants before the commencement of the intervention. The aim, method, benefits, and drawbacks of the study were explained to participants, and they were aware of their right to withdraw from the trial at any phase of intervention.

### 2.2. Participants

Women who met the following inclusion criteria were selected to be enrolled in this project: (1) aged 18–50; (2) normal Pap smear; (3) regular menstrual cycle; (4) endometrial line thickness lower than 10 mm; (5) PBAC (Pictorial Blood Loss Assessment Chart) score more than 100; (6) normal gynecological observation; and (7) menstrual period longer than 7 days.

They were not included in the study if they were pregnant, breastfeeding, or wanted to become pregnant during the next 3 months, experienced acute conditions, including hypertension, diabetes, liver or kidney diseases, coagulopathies, thyroid dysfunctions, and chronic inflammatory diseases, involved in endometrial abnormalities (hyperplasia, cancers of cervix, uterine or ovarian, endometriosis, submucosal or intramural fibroids larger than 4 cm, and pelvic inflammatory diseases), had hemoglobin less than 10 mg/dl, and treated with hormonal medications and antifibrinolytic drugs. Patients who did not follow the treatment regimen, did not have an interest in the continuing study, did not tolerate the medications, or required emergency procedures due to intensified bleeding were excluded during the intervention.

### 2.3. Sample Size

The following formula was used to compute the required sample size. A total of 40 participants per group were estimated considering type I error of 0.05, study power of 80%, attrition rate of 10%, standard deviation = 144 and 90 in each group, and difference of PBAC between groups = 80.

(1) $$\begin{array}{c} n=\frac{\left(\sigma^{2}\right){\left(Z_{\left(a/2\right)}\right)}^{2}}{{\left(d\right)}^{2}}=36\text{.} \end{array}$$

### 2.4. Randomization and Blinding or Masking

Participants were randomly allocated into one of the groups of TA (*n* = 40) or PG (*n* = 40). To generate a random sequence, the technique of computer-based block randomization was used and the random codes were assigned in a 1 : 1 ratio, with a block size of four. Randomization was done by a researcher who had no clinical role in the trial. Furthermore, other protocols like enrollment, sequence generation, allocation concealment, and randomization process were carried out by the principal investigators.

In order to blind participants to the samples, the capsules in both groups were almost similar. There was no detectable difference in shape, color, and size of capsules and containers in both groups, and they were recognized only through allocated codes. The capsules were coded differently in each group to blind the investigator. In addition, the biostatistician encoded the data for each group to ensure that the data remained masked.

### 2.5. Formulation of Golnar Product and TA

The formulation of this polyherbal medication is based on Exir Azam (Great Elixir) of Mohammad Azam Khan Chasti (18th AD), which is one of the reference books of TPM [24]. An adequate amount of pomegranate flower (*Punica granatum* L.*, Lythraceae*), myrtle (*Myrtus communis* L.*, Myrtaceae*), and Arabic gum (*Acacia senegal* (L.) Willd.*, Leguminosae*) was procured from a traditional herbal market. They were identified and confirmed by a botanist at the Herbarium Center of the School of Pharmacy, Tehran University of Medical Sciences, with voucher numbers PMP-537, PMP-1640, and PMP-893, respectively. The equal amount of three herbs was mixed and pulverized with an electric mill. Then, 500 mg of provided powder was packed in capsules.

Total bacterial counts, as well as specific microbial tests (on *Staphylococcus aureus*, *Pseudomonas aeruginosa*, and yeast) on Golnar powder, were performed, and the results were compared with the pharmacopoeia. The results of the test showed that total bacterial counts were in the normal range.

Total phenolic content was estimated by a spectrophotometer according to the Folin–Ciocalteu method, and absorbance was measured at 765 nm against a prepared blank. Gallic acid was used as a standard to construct the standard curve. The total phenolic content was expressed as mg gallic acid per gram of preparation. The total amount of phenol in 100 mg of the powder was 11.02 ± 0.62 mg gallic acid.

Two types of capsules were prepared as medication in the control group. The first type contained 500 mg tranexamic acid, which is prepared by combining two 250 mg tablets, and the second type contained lactose. There was no detectable difference in shape, color, and size of capsules and containers in both groups, and they were recognized only through allocated codes.

### 2.6. Intervention

The duration of this study included three consecutive menstrual cycles (12 weeks), in which participants in both groups consumed 3 capsules daily (one after each meal, breakfast, lunch, and dinner). Participants in each group received two small and big bottles on the first day of the menstrual cycle. Small bottles comprised 15 capsules, which were used for the first five days of cycles, and there were 75 capsules in big bottles for the rest of the days. The content of all capsules in the intervention group was Golnar product. On the other hand, the 15 capsules in small bottles in the control group contained tranexamic acid, which was used for the first five days of the cycle, and capsules in big bottles contained lactose as a placebo.

After selecting eligible participants and initial assessments, a small bottle and a big one were given to all participants in both groups and were trained on how to consume them. The number of capsules in bottles was enough for a month. Patients were scheduled for monthly appointments to measure outcomes and assess potential side effects. During these visits, they delivered empty bottles and were given new full ones. In addition, participants received a phone call from one of the researchers weekly to check possible adverse effects and they were also reminded to take capsules.

### 2.7. Outcomes

At the baseline of the study, the patients were assessed for demographic characteristics, such as age, body mass index (BMI), marital status, occupation, education, complete history, physical examination, uterine sonography, Pap smear, and complete blood count (CBC). The primary outcome was the amount of bleeding over menstruation evaluated by the Pictorial Blood Loss Assessment Chart (PBAC). Secondary outcomes were premenstrual symptoms, quality of life, and serum level of hemoglobin.

The Pictorial Blood Loss Assessment Chart (PBAC) which is a semi-quantitive tool was used to measure the amount of blood loss over menstruation. This chart was introduced by Higham et al. and is scored based on the visual appearance of the number of sanitary products used, the number and size of blood clots, and the number of menstrual days. This tool was filled by women at the beginning of the study and after three consecutive cycles [25].

The Premenstrual Symptoms Screening Tool (PSST) [26] and menorrhagia questionnaire (MQ) [27] were other instruments that were used to evaluate the severity of premenstrual dysphoric disorder (PMDD) and quality of life, respectively. MQ is a 13-item questionnaire, and each respondent gets a score between 0 and 100, in which a higher MQ score indicates lower quality of life. In addition, the PSST instrument includes 19 questions, and the score of each question ranges from 0 (not applied) to 3 (severe). These questionnaires were completed by participants at the baseline and the end of the study.

Moreover, a blood sample was taken from patients twice (at the commencement and completion of the study) to measure hemoglobin levels. A checklist derived from the Common Terminology Criteria for Adverse Events (CTCAE) was used to assess the potential side effects.

A schedule of study procedures is presented in Table 1.

### 2.8. Statistical Analyses

As the aim was to investigate the equivalence of two treatments, the hypothesis of difference between the treatments should have been rejected. For baseline characteristic assessment, categorical variables were reported as a percentage and were analyzed using the chi-square test, while continuous variables were expressed as mean ± standard deviation (SD) and compared between groups using Student's *t*-test. The per-protocol method was used to analyze primary and secondary outcomes. PBAC outcomes, including the number of pads, clot number, and the number of menstrual days, were compared between groups over a period. For the MQ score, PMDD score, and hemoglobin variables, Student's *t*-test and paired *t*-test were used to compare between groups and measure the changes of variables in each group, respectively. A chi-square test was used to evaluate side effects between groups. A significance level was determined at lower than 0.05. All statistical analysis was carried out by R software version 3.6.0.

## 3. Results

**Figure 1:**
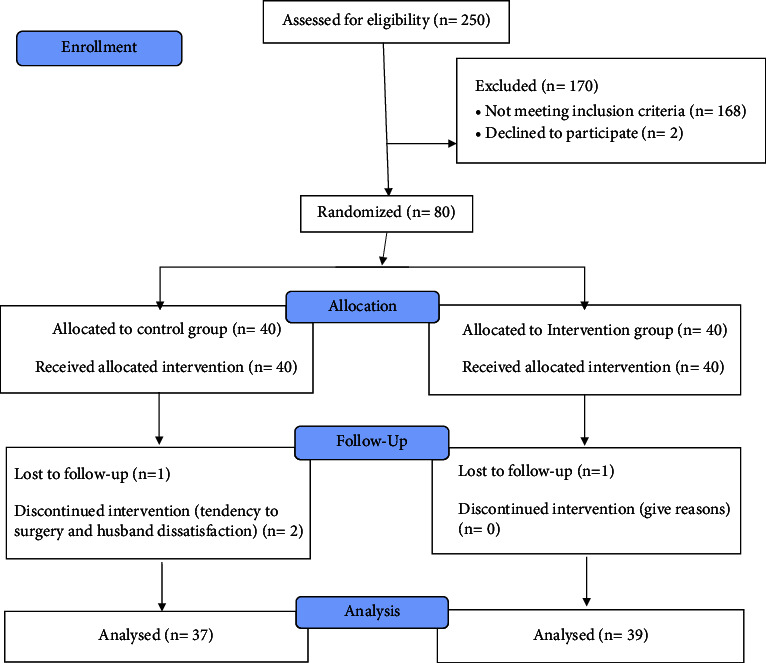
Flow diagram of the study.

The flow diagram of the study is indicated in Figure 1. Among 250 attendees examined for eligibility, eighty of them who met the inclusion criteria were selected and randomized into PG (*n* = 40) and TA (*n* = 40) groups. At the end of the follow-up, four patients (3 in the TA group and 1 in the Golnar group) withdrew and 76 participants completed the trial. The general characteristics of the participants are described in Table 2. No significant difference was observed between the two groups in terms of baseline characteristics.

**Figure 2:**
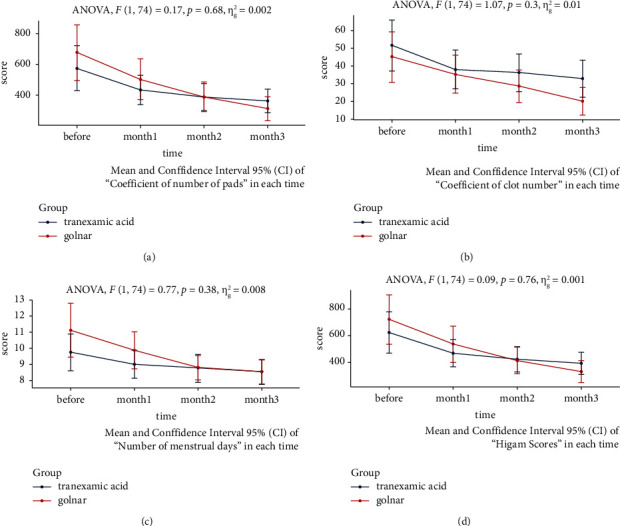
Repeated measure ANOVA analysis for assessing the number of pads (a), clot number (b), number of menstrual days (c), and Higham score between groups (d).

The results of repeated measure ANOVA analysis showed that there was no significant difference between the two groups at the baseline of the study in the subscales and overall score of PBAC. In addition, although the mean score of participants in both groups decreased gradually and significantly over three measurements during and end of the study, no considerable difference was observed between the PG and TA groups in any measurements (Figure 2).

The mean scores of MQ, PMDD, and hemoglobin, at the first and end of the study, in both TA and the Golnar groups are presented in Table 3. Results demonstrated that no considerable differences in any scales between the two groups were observed at the study's baseline (*P* > 0.05). At the end of the study, differences in PMDD score and hemoglobin between groups were still non-significant, while participants in the PG group had significantly lower MQ score compared to those who were in the TA group. In intra-group comparison, all variables changed significantly (*P* ≤ 0.001), except for hemoglobin in the TA group (*P* = 0.579).

There was no significant difference in terms of side effects except constipation that participants in the TA group experienced more (Table 4).

## 4. Discussion

The results of the current study indicated that the Golnar product is an effective complementary medicine in the treatment of AUB without notable side effects. This polyherbal product can lower bleeding volume and improve the quality of life and symptoms of PMDD as efficacious as tranexamic acid as a standard treatment. In addition, hemoglobin levels increased significantly in patients who consumed the Golnar product, while it did not change in the TA group.

Treatment satisfaction and safety of patients are principal reasons for the reappraisal of conventional therapies of AUB [28]. The results of the present study indicated that PG and TA both decreased the amount of bleeding over menstruation cycles, while there was no significant difference between them neither before nor after the intervention. The antihemorrhagic effects of the ingredients of this product have been shown in previous studies. The results of the present study were consistent with a study conducted by Goshtasebi and collaborators [20]. They concluded that intervention with TA or pomegranate flower in the first 5 days of the menstrual cycle for three sequential months could equally mitigate the PBAC score. In addition, a trial by Qaraaty et al. [23] indicated that a 15 ml intake of myrtle syrup at the first 7 days of the cycle for three consecutive menstrual periods declined bleeding compared to a placebo. The hypothesis was that co-administration of pomegranate flower and myrtle which are components of Golnar product with longer duration can induce more therapeutic effects.

Furthermore, similar effects have been observed in a few studies on menorrhagia [18, 21, 29]. In the study of Memarzadeh et al. which was a before/after trial, the effects of the pomegranate flower were evaluated solely. The final findings approved that pomegranate syrup is an effective treatment for the reduction of bleeding induced by uterine leiomyoma [29]. In another trial, the efficacy of pomegranate peel in the improvement of menorrhagia was equal to mefenamic acid [21]. Moreover, similar to the current study, Yousefi et al. used a multiherbal product, in which pomegranate flower was the main ingredient, for the treatment of heavy menstrual bleeding. Finally, they showed that herbal drugs significantly decreased PABC score in comparison with placebo [18]. In line with current intervention, some investigations indicated that herbal therapy with PG ingredients is effective in the improvement of blood hemoglobin which is the consequence of bleeding reduction [18, 20, 21, 29].

In addition, participants in both groups of PG and TA had lower scores in MQ and PMDD score after intervention in comparison with their baseline values. While the difference in baseline values was not significant between groups for variables of quality of life and PMS, final scores indicated that patients in the PG group experienced better quality of life compared to the TA group. Some studies indicated that AUB has detrimental effects on patients' quality of life [30, 31]. Therefore, it seems that treatment of heavy menstrual bleeding could improve quality of life. Several studies have reported an improvement in quality of life after consumption of Golnar product and its components [18, 20, 21, 23, 29]. Other studies also demonstrated that pomegranate peel extract or supplements could improve the quality of life of hemodialysis patients and menopausal women [32, 33].

On the other hand, premenstrual disorders are destructive factors for women's quality of life [34, 35]. Thus, any improvement in PMDD status could result in higher life satisfaction. In addition, a review by Guerrero-Solano and colleagues reported that some compounds in pomegranate have pain reliever effects, particularly pains with inflammatory and nociceptive origin, which are considered a common feature of PMS [36, 37].

Pomegranate as a herbal medicine has beneficial effects on a variety of other conditions such as diabetes, cardiovascular diseases, cancer, arthritis, Alzheimer's, infertility, diarrhea, and vaginal discharge [38, 39]. Different parts of the pomegranate plant, including roots, bark, leaves, fruits, and flowers, have been traditionally applied [40]. It contains several bioactive compounds like quercetin, ellagic acid, punicalagin, pedunculagin, tannic acid, anthocyanins, rutin, catechin, and polyphenols, which show anti-inflammatory, antioxidant, antiangiogenic, anticancerous, antimutagenic, cytoprotective, cardiovascular protective, antidiabetic, antiulcerogenic, and blood tonic potentials [39]. In addition, a study by Esawy et al. indicated that mouthwash containing *Punica granatum* peel crude extract could reduce the clotting time which proves its antihemorrhagic activities [41].

The exact mechanism of how the pomegranate flower help mitigates bleeding is not known, but it is apparently due to its anti-inflammatory and antioxidant properties [42]. Modern research has shown the critical role of inflammation in causing AUB [43, 44]. The vasodilatory effects of prostaglandin E2 and I2 as well as the antiplatelet aggregation activity of prostaglandin I2 play a significant role in extreme bleeding during menstruation [45]. A study by Xu et al. indicated that 0–100 *μ*g/mL of pomegranate flower declined the production of PGE2, IL-6, IL-1*β*, and TNF-*α* significantly [46]. Moreover, bioactive compounds such as ellagic acid, gallic acid, and punicalagin extracted from pomegranate suppress PGE2 and IL-6 production [47].

This study was conducted with enough sample size, appropriate intervention duration, and a triple-blind design. Nevertheless, there are some potential limitations: (1) patients were not followed after ending the study and (2) laboratory tests that assess anticoagulant factors and kidney and liver function were not conducted.

In conclusion, the findings of the current trial supposed that the Golnar product is as effective as tranexamic acid in controlling bleeding and enhancing the quality of life and premenstrual symptoms.

## Figures and Tables

**Table 1:** Schedule of study procedures.

Procedure	Before study	First cycle	Second cycle	Third cycle
Baseline features	×			
Blood loss	×	×	×	×
Premenstrual dysphoric disorder	×			×
Quality of life	×			×
Hemoglobin level	×			×
Side effects	×	×	×	×

**Table 2:** Baseline characteristics of participants.

Variables	Golnar group (*n* = 40)	TA group (*n* = 40)	*P* value
Age (years)	39.83 ± 9.91	38.65 ± 8.42	0.57
BMI (kg/m^2^)	27.29 ± 5.37	25.31 ± 5.38	0.10
Marital status			ns
Single	8 (20%)	10 (25%)	
Married	32 (80%)	30 (75%)	
Occupation			ns
Housewife	28 (70%)	29 (72%)	
Employed	12 (30%)	11 (28%)	
Education			ns
≤Diploma	19 (47%)	17 (42%)	
License	15 (38%)	16 (40%)	
>License	6 (15%)	7 (18%)	

Categorical variables are represented as percentages (%) and continuous data are represented as mean ± SD. ^a^*T*-test. ^b^Chi-square for trend test. _a_: age and BMI; _b_: marital status, occupation, and education.

**Table 3:** Results of MQ score, PMDD score, and hemoglobin between intervention and control groups.

Variables	Golnar group (*n* = 39)	TA group (*n* = 37)	*P*value^a^
	Mean ± SD	Mean ± SD	
MQ score			
Pretreatment	64.23 ± 19.91	60.22 **±** 17.40	0.355
Posttreatment	33.41 ± 17.18	46.67 ± 18.89	0.002
*P* value^b^	<0.001	<0.001	
PMDD score			
Pretreatment	22.79 ± 15.90	19.40 ± 13.58	0.322
Posttreatment	12.82 ± 11.64	15.73 ± 12.94	0.306
*P* value	<0.001	<0.001	
Hemoglobin (g/dl)			
Pretreatment	11.76 ± 1.60	12.33 ± 1.43	0.111
Posttreatment	12.16 ± 1.54	12.28 ± 1.54	0.735
*P* value	0.001	0.579	

SD, standard deviation; TA, tranexamic acid; MQ, menorrhagia questionnaire; PMDD, premenstrual dysphoric disorder. ^a^*P* value is calculated by the independent *t*-test. ^b^*P* value is calculated by the paired *t*-test.

**Table 4:** Comparison of side effects of treatment in both groups.

Variables	Golnar group	Tranexamic acid group	*P* value^a^
Gastric pain	2	4	0.358
Nausea	1	2	0.525
Diarrhea	1	4	0.147
Constipation	0	4	0.035
Headache	1	1	0.970

^a^Results come from a chi-square test.

## Data Availability

The data used to support the findings of this study are available from the corresponding author upon request.
